# Publisher Correction: Seasonal synchronization of sleep timing in industrial and pre-industrial societies

**DOI:** 10.1038/s41598-019-51010-5

**Published:** 2019-10-08

**Authors:** José María Martín-Olalla

**Affiliations:** Universidad de Sevilla, Facultad de Física, Departamento de Física de la Materia Condensada, ES41012 Seville, Spain

Correction to: *Scientific Reports* 10.1038/s41598-019-43220-8, published online 01 May 2019

In this Article there is an error in Figure 1 where the map is truncated. The correct Figure 1 appears below.

**Figure 1:**
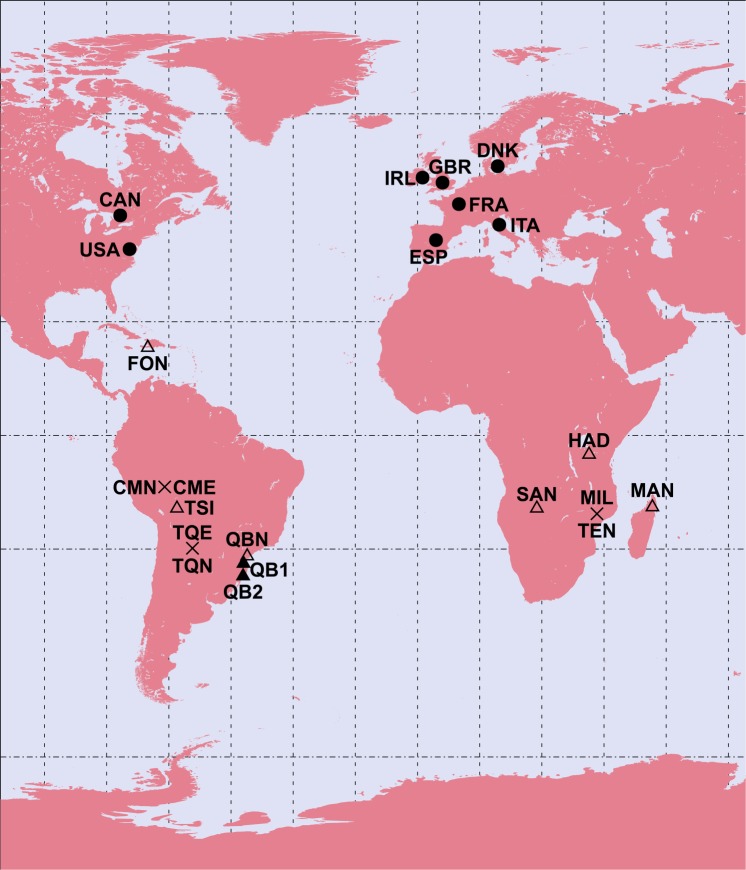
.

