# Supplementary material for: Seasonal synchronization of sleep timing in industrial and pre-industrial societies
Source: Sci Rep. 2019 May 1;9:6772. doi: 10.1038/s41598-019-43220-8 (PMC6494851; doi:10.1038/s41598-019-43220-8)
Supplement: Supplementary file 1 — Supplementary material [file 41598_2019_43220_MOESM1_ESM.pdf]

Supplementary material to: “Seasonal synchronization of sleep timing in industrial and pre-industrial societies”

José María Martín-Olalla[1]

[1]Universidad de Sevilla, Facultad de Física. Departamento de Física de la Materia Condensada. ES41080 Seville, Spain
[*]email: olalla@us.es; twitter: @MartinOlalla_JM

ABSTRACT

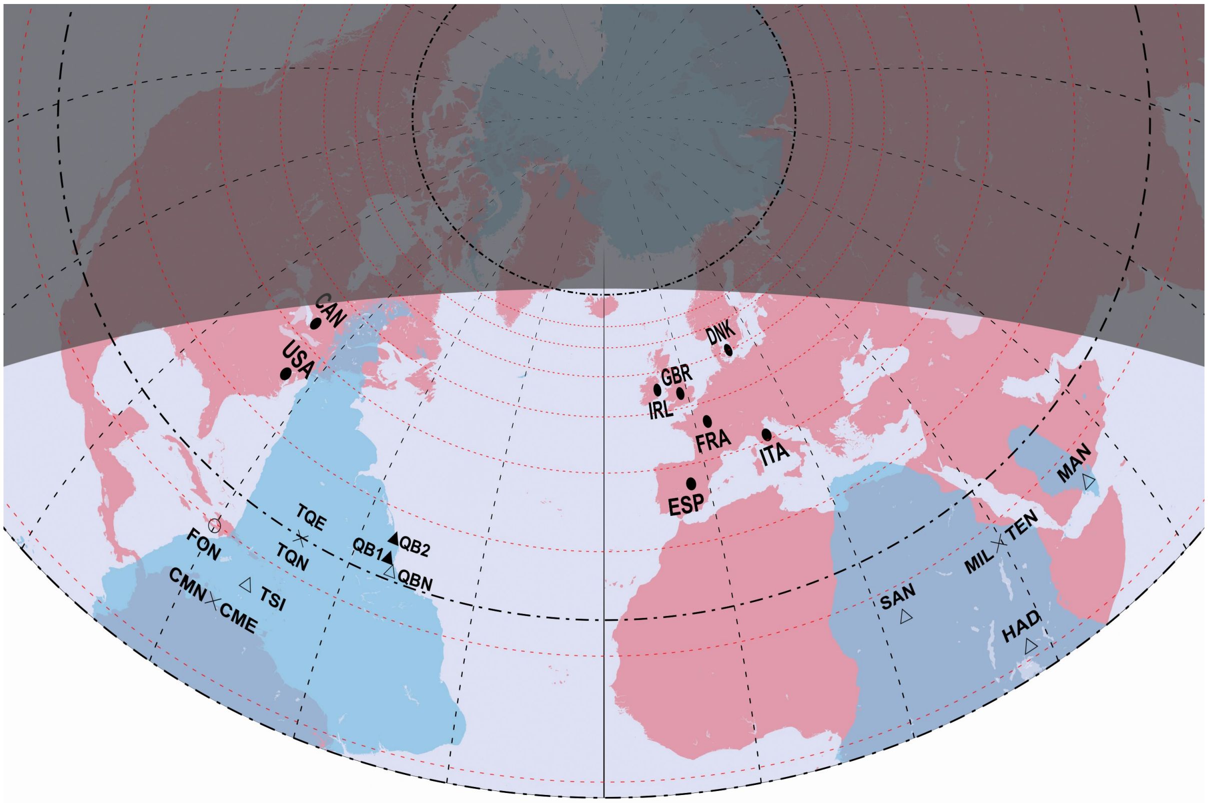

Figure S1. Locations of the societies whose sleep timing is analyzed in this manuscript on an azimuthal projection centered at $\lambda_0 = 18°8'$ west of UTC prime meridian and $52°$ latitude. The picture shows the Earth on a unique hemisphere from Equator (bottom) to the Pole (top). Solid symbols display societies with access to electricity. Open symbols display societies without access to electricity. Crosses display pairs of pre-industrial societies with and without access to electricity. Rounded symbols refer to societies physically in the Northern Hemisphere. Rest of symbols locate societies physically in the Southern Hemisphere. Circles of latitude in heavy dash-dotted lines are the Equator, the Tropic (at $\phi = \varepsilon$) and the Polar Circle (at $\phi = 90° - \varepsilon$). Circles of latitude in light dashed lines run values of the shortest photoperiod D_w, increasing in steps of 1 h; the circle nearest to the Polar Circle is $D_w = 5$ h. The picture separates light and dark in the winter day at noon over the meridian λ_0. Continental and lake polygons were taken from http://www.naturalearthdata.com/.
